# Elevational Distribution and Conservation Biogeography of Phanaeine Dung Beetles (Coleoptera: Scarabaeinae) in Bolivia

**DOI:** 10.1371/journal.pone.0064963

**Published:** 2013-05-22

**Authors:** Sebastian K. Herzog, A. Caroli Hamel-Leigue, Trond H. Larsen, Darren J. Mann, Rodrigo W. Soria-Auza, Bruce D. Gill, W. D. Edmonds, Sacha Spector

**Affiliations:** 1 Asociación Armonía, Santa Cruz de la Sierra, Bolivia; 2 Museo de Historia Natural Alcide d’Orbigny, Cochabamba, Bolivia; 3 The Betty and Gordon Moore Center for Ecosystem Science and Economics, Conservation International, Arlington, Virginia, United States of America; 4 Hope Entomological Collections, Oxford University Museum of Natural History, Oxford, United Kingdom; 5 Entomology Unit, Ottawa Plant Laboratory, Canadian Food Inspection Agency, Ottawa, Canada; 6 Marfa, Texas, United States of America; 7 American Museum of Natural History, Center for Biodiversity and Conservation, New York, New York, United States of America; Field Museum of Natural History, United States of America

## Abstract

Insect macroecology and conservation biogeography studies are disproportionately scarce, especially in the Neotropics. Dung beetles are an ideal focal taxon for biodiversity research and conservation. Using distribution and body size data on the ecologically important Phanaeini, the best-known Neotropical dung beetle tribe, we determined elevational patterns of species richness, endemism, body size, and elevational range in Bolivia, specifically testing Bergmann’s and Rapoport’s rule. Richness of all 39 species and of 15 ecoregional endemics showed a hump-shaped pattern peaking at 400 m, but overall declined strongly with elevation up to 4000 m. The relationship between endemic and total species richness appeared to be curvilinear, providing only partial support for the null hypothesis that species-rich areas are more likely to be centers of endemism by chance alone. An elevational increase in the proportion of ecoregional endemics suggests that deterministic factors also appear to influence endemism in the Andes. When controlling for the effect of area using different species-area relationships, the statistically significant richness peak became more pronounced and shifted upslope to 750 m. Larger species did not have higher elevational mid-points, and mean body size decreased significantly with elevation, contradicting Bergmann’s rule. Rapoport’s rule was supported: species with higher elevational mid-points had broader elevational ranges, and mean elevational range increased significantly with elevation. The elevational decrease of phanaeine richness is in accordance with studies that demonstrated the combined influence of temperature and water availability on species diversity, but also is consistent with niche conservatism. For invertebrates, confirmation of Rapoport’s and refutation of Bergmann’s rule appear to be scale-invariant general patterns. Analyses of biogeographic patterns across elevational gradients can provide important insights for identifying conservation priorities. Phanaeines with narrow elevational ranges on isolated low-elevation mountains in eastern Bolivia are at greatest climate-change related extinction risk from range-shift gaps and mountaintop extinctions.

## Introduction

After several decades of research elucidating patterns of species richness along elevational gradients (e.g., [1]–[6]), in recent years much emphasis has been placed on developing a comprehensive understanding of these patterns and their underlying causes [7]–[15]. Most commonly, the elevational pattern of species richness is hump-shaped with maximum richness at some intermediate point of the gradient [2], [9], [16]. By contrast, elevational patterns of other biogeographic parameters such as body size distributions (e.g., [17]) or endemism (e.g., [18], [19]) have received considerably less and more recent attention. This is particularly the case for invertebrates, which comprise the vast majority of known biodiversity on Earth. However, macroecological patterns of insects are poorly documented [20], especially in biodiversity hotspots [21].

Two prominent biogeographic hypotheses have been proposed along elevational gradients. Bergmann’s rule [22] has been the subject of recurring debate with respect to its precise definition, metabolic scope (endotherms *versus* homeotherms *versus* ectotherms) and taxonomic level (intraspecific *versus* interspecific), and whether or not it inherently implies a mechanism (see [23], [24] and references therein). Meiri [24] defined Bergmann’s rule in a broad sense as ‘a tendency of organisms to be smaller at high temperatures and low latitudes and larger at low temperatures and high latitudes’ and argued that it is a pattern that can be studied regardless of mechanism in any taxon and at any taxonomic level, which we follow here. Bergmann’s rule was applied to elevational gradients by Hawkins and DeVries [25] and more explicitly by Brehm and Fiedler [17], predicting that animal body size increases with elevation.

Rapoport’s elevational rule [26] predicts that species at higher elevations have greater elevational ranges. According to this hypothesis, which is also known as the climatic variability hypothesis (see [27]), species at higher elevations can tolerate greater climatic variability and therefore have larger elevational ranges, because high-elevation climates are more variable and show a greater magnitude of extremes than low-elevation climates.

Whether either rule applies to insects and other invertebrates along elevational gradients has only been tested by a handful of studies, especially in the highly diverse Neotropics. Moreover, no convincing general hypothesis that explains insect body size patterns along climatic gradients is currently available [17]. At the interspecific or assemblage level, Bergmann’s rule was not supported by studies on Neotropical lepidopterans [17], [25] and flies [28], nor for European land snails [29]. Rapoport’s rule, on the other hand, has been found to apply to a small number of insect and arachnid taxa in South and Middle America [28], [30], [31], North America [32], [33], Africa [27] and Europe [34], [35]. Only Kubota et al. [28] tested both rules for the same taxonomic group (tephritid flies) and data set in southeast Brazil, albeit across a relatively narrow elevational gradient (700–2500 m). Thus, additional studies on a broader range of taxa are needed to determine whether these emerging biogeographic patterns generally apply to invertebrates [17]. Further, it is unknown whether these patterns are scale-dependent, as is the case for macroecological patterns of species richness [9], [36], because almost all of the above studies were conducted at local spatial scales.

Scarabaeine dung beetles (Coleoptera: Scarabaeidae) are an ideal focal taxon for biodiversity research and conservation [37], [38], [39]. The monophyletic Phanaeini (ca. 160 species) are the taxonomically and biogeographically best-known scarabaeine dung beetle tribe in the Neotropics (see [40]–[45]). They are endemic to the Americas [46] and are largely comprised of tunnellers [44], [47] that bury dung in tunnels excavated directly below droppings. Due to their comparatively large size they are likely to be particularly important for ecosystem functioning [48] and ecological processes such as secondary seed dispersal [49]. An extensive review of the distribution and natural history of phanaeines in Bolivia reported the occurrence of 39 species in the country [50], [51].

In the present study we assessed country-level elevational patterns of total (assemblage) species richness, species richness and proportion of ecoregional endemics, body size and elevational range amplitude for Bolivian phanaeines. The elevational pattern in total species richness was determined controlling for the confounding effect of land surface area following Rahbek’s [3] approach. With respect to the elevational pattern of ecoregional endemics, we tested the null hypothesis that species-rich areas are more likely to be centers of endemism by chance alone [52] (see also [53]). Whether Bergmann’s and Rapoport’s rule apply to phanaeines in Bolivia was tested at the species and species assemblage (elevational zones) levels. At the species level, Bergmann’s rule predicts a significant positive relationship between a species’ mean body size and the mid-point of its elevational range, whereas Rapoport’s rule predicts a significant positive relationship between the elevational mid-point and elevational range of species. For species assemblages across elevational zones, Bergmann’s rule predicts that the mean body size of assemblages increases with elevation, whereas Rapoport’s rule predicts that the mean elevational range of assembages increases with elevation. Understanding the influence of elevation on species distributions and endemism is a key issue in the newly emerging field of conservation biogeography [20], [54], particularly for tropical ectotherms, due to their high sensitivity and vulnerability to climate change [55], [56], [57]. We discuss the implications of our findings for conservation planning.

## Materials and Methods

### Study Area and Data Set

Bolivia covers an area of 1 098 581 km^2^ with an elevational gradient that ranges from about 80 m in the eastern lowlands to 6542 m in the Andes in the southwest of the country. It is located on the transition from tropical to subtropical regions spanning a 1460-km latitudinal gradient from about 9°40’S to 22°52’S, resulting in an ecological division into 12 ecoregions [58], [59].

We compiled a distributional data base of the occurrence of 39 phanaeine species based on 178 georeferenced Bolivian collecting localities [50], [51] (raw distributional data available in Hamel-Leigue et al. [50]); one additional locality could not be georeferenced, but was assigned with certainty to an elevational zone (see below) and ecoregion and included in the analyses. Five localities that could not be georeferenced or assigned with certainty to a given ecoregion due to ambiguous information were excluded. The minimum distance between localities was 1.0 km; collecting sites or transects with a spatial proximity of <1.0 km were combined to form a single locality.

Data sources included literature accounts, unpublished collecting work and reference collections of the authors (which accounted for 89 (50%) of the 179 localities), and specimens in six museums reviewed by ACHL, DJM and THL (see [50], [60] for details). Geographic coordinates and elevation of localities sampled by the authors were determined in the field using hand-held GPS units and, in some cases, altimeters. Museum specimen and literature localities lacking specific coordinates or elevation were georeferenced based on the site description provided, using topographic maps, gazetteers (e.g., [61]) and Google™ Earth. Elevations of all localities were verified with Google^™^ Earth.

### Species Richness, Endemism and Elevational Distribution

To examine elevational patterns we rounded locality elevations to the nearest 50 m, calculated the elevational range amplitude and mid-point for each species and determined the presence of species in elevational zones of 250 m (0–249 m, 250–499 m, and so on, following [8]). For species with apparent gaps in their known elevational distribution we used interpolation, under the assumption that each species is distributed continuously between its recorded upper and lower limit [62]. See Table S1 for the elevational limits of each species. As an estimate of the level of endemism we examined the elevational richness pattern in absolute and relative terms of those 15 species that are endemic to a single ecoregion (Table S1; see [51] for details) based on the ecoregion classification of Ibisch et al. [59].

The elevational distribution of phanaeine collecting localities is shown in Table 1. Because some data sources (especially museum collections) are likely to provide information only on species presence, possibly resulting in false-absence data, we determined the number of localities per elevational zone that were inventoried systematically using pitfall trap transects (≥10 traps run for ≥3 days) or by intensive manual collecting at dung pats (≥10 dung pats across an area of ≥1 ha) *versus* localities with only opportunistic manual collecting or without information on collecting methods (Table 1). Some elevational sampling biases are evident in Table 1, particularly with respect to the number of systematic inventories in the five highest elevational zones (≥2750 m). However, six additional localities >3000 m were inventoried systematically by the authors, but no phanaeine dung beetles were recorded at these sites. More importantly, interpolation of species occurrences across apparent elevational range gaps can largely be expected to account for incomplete sample coverage. In addition, Hamel-Leigue et al. [51] did not find serious geographic sample coverage bias in our data set based on ecoregions as units of analysis. Thus, we consider that our interpolated data set is not substantially biased by incomplete sample coverage or sampling effort.

**Table 1:** Distribution of 179 Bolivian phanaeine dung beetle collecting localities by elevational zone.

Elevation (m)	Systematic inventories (*N* = 109)	Opportunistic collecting (*N* = 70)	Total
0–249	39	15	54
250–499	20	16	36
500–749	16	15	31
750–999	10	4	14
1000–1249	6	0	6
1250–1499	6	1	7
1500–1749	3	3	6
1750–1999	3	5	8
2000–2249	2	1	3
2250–2499	1	2	3
2500–2749	4	4	8
2750–2999	0	2	2
3000–3249	0	0	0
3250–3499	1	1	2
3500–3749	1	3	4
3750–3999	1	2	3

Localities with an elevational range that extended across two elevational zones, or with an elevation that corresponds to the limit between two zones, were assigned to both zones. Six additional localities >3000 m were inventoried systematically by the authors, but no phanaeine dung beetles were recorded at these sites. No species is known to occur >4000 m in Bolivia [46], [47].

### Body Size

We used length (measured in dorsal aspect from pygidium to anterior margin of clypeus), which is highly correlated with width and depth (SKH and ACHL, unpubl. data), as a measure of body size because it is the most accurate predictor of biomass in dung beetles [63]. For 24 species we measured (to the nearest 0.1 mm) between 6 and 107 specimens per species using digital calipers, for an approximately equal number of males and females, and determined the arithmetic mean for each species (Table S1). For 15 species with <6 specimens available to us (Table S1) we also obtained length values from the literature [40]–[43], [64]–[67] (most sources did not provide information on the sex of the individuals measured) and used the mid-point between the minimum and maximum value (rounded to the nearest 0.5 mm) for each species as a proxy for the arithmetic mean. Based on individual species’ means we determined the mean body size of all species (mean of the species means) for each elevational zone.

### Statistical Analyses

To test the null hypothesis that species-rich areas are more likely to be centers of endemism by chance alone, we regressed the number of ecoregional endemics against total species richness in each elevational zone (excluding elevations ≥2000 m, where no endemics were found). As endemic species occur in both the dependent and independent variable, we applied formula 15.10 in Sokal and Rohlf [68] for a part-whole correlation to our data set to determine whether regression results are inflated. The resulting correlation coefficient was identical to the *R* value of the regression, indicating that the latter is not inflated. We also used linear regression to examine the relationship of the proportion of ecoregional endemics with elevation.

To examine the relationship of the number of phanaeine species (per elevational zone) with elevation in Bolivia we followed Rahbeks’s [3] approach to control for the confounding effect of land surface area on species richness (see also [69]). This approach requires the constructing of species-area (SAR) curves, usually based on the Arrhenius [70] equation (e.g., [15]). The major challenge of constructing SAR curves is to obtain realistic values for the slope of the species-area relationship in log-log space (*z*-values). To assess the magnitude and potential general range of *z*-values for South American phanaeines, we compiled species lists for the remaining 12 South American countries based on available information on distributional ranges [40]–[43], [45], [71], [72] for a total of 89 species in 11 genera (Table S2). We then determined empirical *z*-values for three country groupings (see [3] for rationale): (A) all countries except Chile, (B) all countries except Chile and Brazil and (C) only the tropical Andes countries Bolivia, Colombia, Ecuador and Peru.

Taking into account the range covered by the three empirical *z*-values (0.299–0.382), we employed a somewhat wider range of seven *z*-values (0.20, 0.25, 0.30, 0.35, 0.40, 0.45, 0.50) to construct SAR curves for each elevational zone based on the interpolated number of species and area of each zone in Bolivia. Area was determined from NASA’s Shuttle Radar Topography Mission digital elevation model (DEM) (http://www2.jpl.nasa.gov/srtm/). To be able to import the raster layer into Microsoft Excel and count the number of cells in each elevational zone (Table S3), we resampled the DEM to a resolution of 0.08333 arc degree cells (9.27×8.91 km, 82.54 km^2^) using bilinear interpolation in ArcGIS 10.

**Figure 2:**
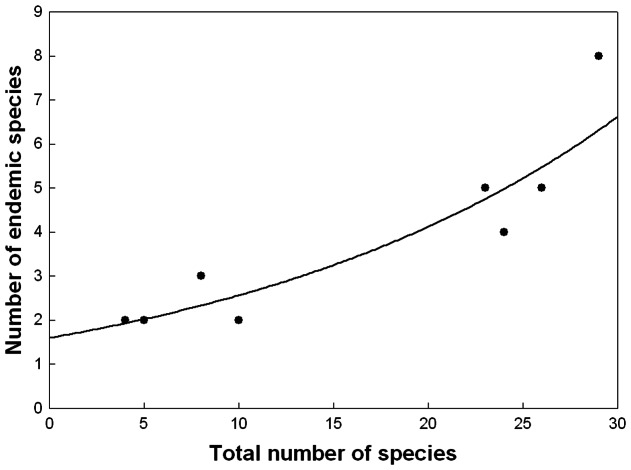
The relationship of ecoregional endemic with total phanaeine species richness. The curve represents a least squares exponential function.

Rahbek [3] found that empirical *z*-values for South American land birds varied with elevation, ranging from 0.09 to 0.26 for six elevational zones and depending on which countries were included (groupings A, B and C above). Unlike the data used by Rahbek [3], available information on the elevational distribution of South American phanaeines outside Bolivia is too incomplete to construct continental-scale SAR curves for different elevational zones. Under the assumption that the elevational pattern of empirical *z*-values reported for birds [3] may also apply to phanaeine dung beetles (differing only in the magnitude of values), we constructed additional SAR curves based on the empirical phanaeine *z*-values for the three country groupings A, B and C (which are identical to those used in Rahbek’s [3] fig. 2) and adjusted for the corresponding elevational variation in empirical *z*-values for birds reported by Rahbek [3]. This rather strong assumption may introduce unknown biases. However, as shown in Results, area-controlled relationships between species richness and elevation based on elevationally constant *z*-values were very similar to those based on elevationally variable *z*-values derived from downscaled patterns of South American land birds, both in shape and absolute values. Hence, inclusion of this informative step of the analysis does not alter the overall result.

Specifically, for grouping A (all countries except Chile), we determined the mean of Rahbek’s [3] six *z*-values (0.192), which was considered equivalent to our *z*-value of 0.382 for the same grouping. Accordingly, Rahbek’s [3] *z*-values for individual elevational zones were multiplied by 1.993 to obtain the corresponding values for phanaeine dung beetles. The same procedure was used for groupings B and C and their respective *z*-values. Because Rahbek’s [3] elevational zones (0–500 m, 500–1000 m, 1000–1500 m, 1500–2000 m, 2000–3000 m, 3000–4000 m) were wider than those used in the present study, his *z*-values had to be scaled down to 250-m bands. To do so, for each country grouping we plotted the six *z*-values against elevation (placing each data point at the middle of its respective elevational zone) and fitted a curve using spline interpolation. The *z*-value for each 250-m elevational band was then taken from the fitted curve (e.g., at 375 m for the 250–499 m zone). This procedure returned values for 13 out of the 16 elevational zones (Table S4), excluding the lowest (because Rahbek’s [3] lowest-elevation *z*-value was placed at 250 m) and the two highest (because Rahbek’s [3] highest-elevation *z*-value was placed at 3500 m) zones.

Thus, we constructed a total of 10 SAR curves (7 with elevationally constant, 3 with elevationally variable *z*-values) for each of 13 elevational zones (250–3499 m) and 7 SAR curves (with elevationally constant *z*-values) for each of the remaining three zones (0–249 m, 3500–3999 m) based on the interpolated number of phanaeine species and area of each elevational zone. To obtain area-corrected relationships between species richness and elevation we set area to 50 000 km^2^ (see [73] for rationale) in each elevational zone and plotted the corresponding species richness values against elevation, resulting in 10 curves: 7 based on constant *z*-values for the entire elevational range (0–3999 m) and 3 based on elevationally variable *z*-values for 13 zones (250–3499 m). One-way ANOVA and pairwise Tukey tests between elevational zones <2000 m were computed in STATISTICA 7 [74] to determine whether the observed species richness peak was significant.

Whether Bergmann’s or Rapoport’s rule apply to Bolivian phanaeines was tested at the species and species-assemblage (elevational zone) levels. At the species level, we used Pearson correlations to determine the relationship between body size (species means) and elevational mid-point (Bergmann’s rule) and between elevational mid-point and elevational range (Rapoport’s rule). At the assemblage level, we used linear regression to examine the relationship of mean body size (Bergmann’s rule) and mean elevational range (Rapoport’s rule) with elevation. To examine whether the observed patterns may be influenced by phylogenetic constraints, we conducted the analyses for Bergmann’s and Rapoport’s rule separately for the most species-rich genus, *Coprophanaeus* (11 species). Correlation and regression analyses were performed in STATISTICA 7 [74].

Because the assemblage-level regression analyses are affected by spatial autocorrelation, resulting in inflated regression coefficients and significance values, we additionally computed spatial autoregressive models using SAM 4.0 [75]. Discrete localities with geographic coordinates are required for computing these models, precluding use of our interpolated data set of species presence per elevational zone. Instead, we used only a subset of data from the 109 systematically inventoried localities (Table 1) and their respective elevations. Due to some elevational sampling biases of this subset, particularly low sample coverage ≥2750 m (see above), results from this analysis also require cautious interpretation. For each of the 109 localities, we determined the mean body size (Bergmann’s rule) and mean elevational range (Rapoport’s rule) of the phanaeine species recorded. We used simultaneous autoregression based on geographical distances between localities with mean body size or mean elevational range as response variables and elevation as predictor variable. In order not to restrict the analysis to nearest neighbor effects, we also computed regressions using the Gabriel Criterion for creating a connectivity matrix (*N* = 158 connections, mean distance ± SD  = 48.38±53.54 km).

## Results

### Species Richness and Endemism

**Figure 1:**
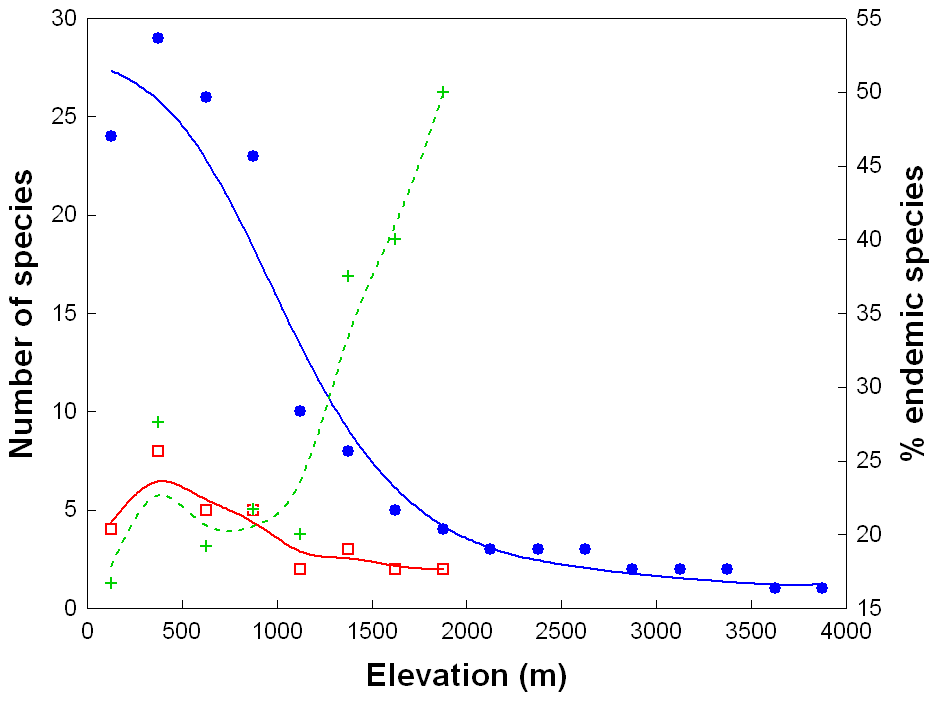
The relationship of total phanaeine species richness (blue dots/solid line), species richness of ecoregional endemics (red squares/solid line) and proportion of ecoregional endemics (green crosses/dashed line) with elevation. Curves were fitted by distance-weighted least squares smoothing.

The elevational distribution of species richness showed a slightly hump-shaped pattern with a peak of 29 species (74% of all Bolivian phanaeines) at 250–499 m (Fig. 1). Species richness decreased sharply around 1000 m, from 23 at 750–999 m to 10 at 1000–1249 m (Fig. 1), corresponding to a 57% decrease. Above 1000 m species richness declined steadily and almost linearly up to 4000 m, above which no species were recorded (Fig. 1). Only 12 species (31%) were recorded regularly above 1000 m, and only 3 (8%) regularly above 2000 m (Table S1). The elevational richness gradient of 15 ecoregional endemics also showed a slightly hump-shaped pattern with a peak of eight species at 250–499 m, and none were recorded above 2000 m (Fig. 1).

The null hypothesis that species-rich areas are more likely to be centers of endemism by chance alone could not be rejected as total species richness was a signifiant predictor of the number of ecoregional endemics per elevational zone (ordinary least squares (OLS) regression: *R* ^2^ = 0.79, *P*<0.01; excluding elevations ≥2000 m). Nonetheless, despite a minor peak at 250–499 m (Fig. 1), the proportion of ecoregional endemics showed an overall increase with elevation (OLS regression: *R* ^2^ = 0.71, *P*<0.01), particularly so above 1000 m, in remarkable contrast to endemic (and total) species richness (Fig. 1). An examination of the residual plot of the regression of endemic against total species richness indicated that the distribution of residuals may be non-random, suggesting a curvilinear relationship, although the low number of data points (*N* = 8 elevational zones) and the lack of total richness values between 10 and 23 species precluded a definite appraisal. Nonetheless, a curvilinear relationship would be concordant with an increase in the proportion of endemics with elevation, and an exponential function appears to provide an appropriate fit for the data (Fig. 2).

**Figure 3:**
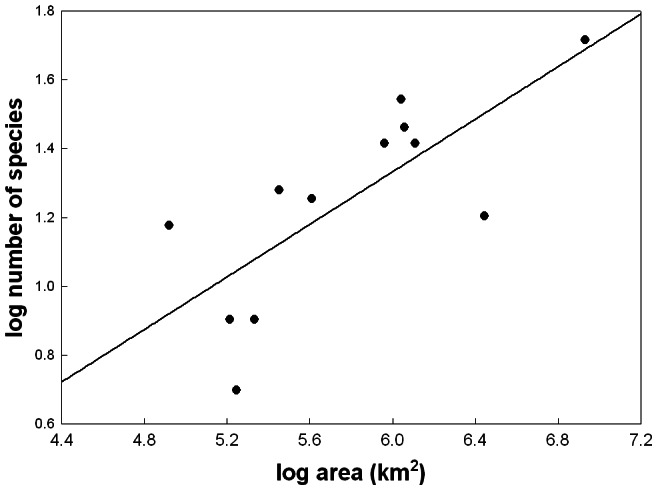
The continental species-area relationship for South American phanaeine dung beetles. Eighty-nine species in 11 genera were included. Each dot corresponds to one of 12 countries (no species have been reported for Chile). See Table S2 for details.

**Figure 4:**
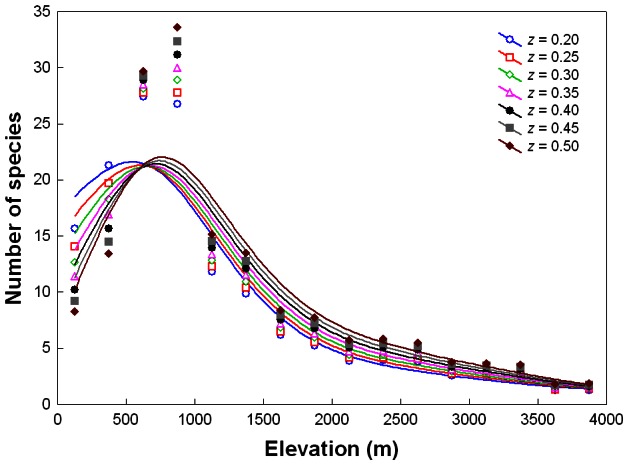
Area-controlled relationships between phanaeine species richness and elevation for different slopes (*z*-values) of the species-area relationship in log-log space. Curves are based on the interpolated number of species and area of each elevational zone in Bolivia, setting area to 50 000 km^2^. Curves were fitted by distance-weighted least squares smoothing.

**Figure 5:**
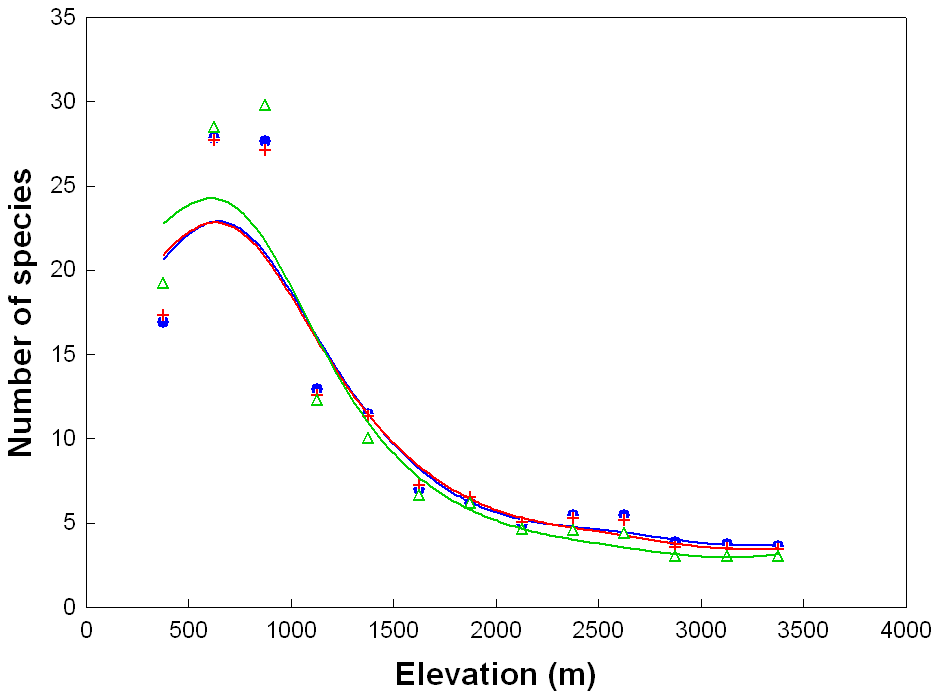
Area-controlled relationships between phanaeine species richness and elevation for elevationally variable *z*-values based on empirical phanaeine *z*-values adjusted for the corresponding elevational variation in empirical *z*-values for birds reported by Rahbek [**3**] **.** Blue dots/line: all South American countries except Chile (grouping A), *z* = 0.382. Red crosses/line: all South American countries except Chile and Brazil (grouping B), *z* = 0.367. Green triangles/line: only the tropical Andes countries Bolivia, Colombia, Ecuador and Peru (grouping C), *z* = 0.299. Curves are based on the interpolated number of species and area of each elevational zone in Bolivia, setting area to 50 000 km^2^. See Materials and Methods for details. Curves were fitted by distance-weighted least squares smoothing.

**Figure 6:**
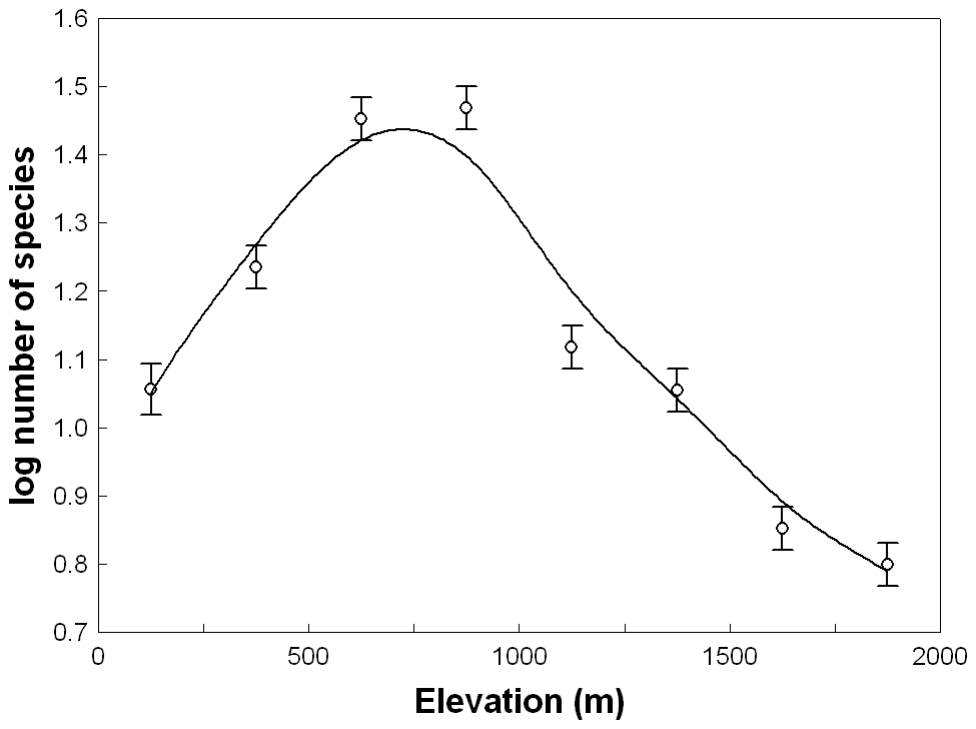
Generalized area-controlled relationship between phanaeine species richness and elevation below 2000 m. Data points represent averages of curves in Figures 3 and 4. Vertical bars denote 95% confidence intervals of a one-way ANOVA (*F* = 244.04, *P*<0.0001). The curve was fitted by distance-weighted least squares smoothing.

The slope of the species-area relationship for South American phanaeine dung beetles varied from *z* = 0.382 for all countries except Chile (grouping A: *R* = 0.75, *P*<0.01; Fig. 3) to *z* = 0.367 for all countries except Chile and Brazil (grouping B: *R* = 0.65, *P*<0.05; data not shown) to *z* = 0.299 for the four tropical Andean countries Bolivia, Colombia, Ecuador and Peru (grouping C: *R* = 0.83, *P* = 0.17; data not shown). Area-controlled relationships between species richness and elevation were rather similar overall for different elevationally constant *z*-values (Fig. 4) and for elevationally variable *z*-values derived from downscaled patterns of South American land birds (Fig. 5). In all cases area correction lead to a more pronounced hump combined with an upslope shift of the species richness peak to about 600–800 m (Figs. 4, 5). Overall, differences in species richness between elevational zones <2000 m were highly significant (ANOVA, *F* = 244.04, *P*<0.0001). Pairwise Tukey tests revealed that the average richness peak at ca. 750 m was significantly higher than richness in the two lower and in all higher zones (Fig. 6, Table 2). The elevational zones 0–249 m, 1000–1249 m and 1250–1499 m did not differ signficantly in species richness (Fig. 6, Table 2). Pairwise Bonferroni corrections with probabilities adjusted for 28 pairwise comparisons did not alter the significance level (i.e., *P*<0.001) of significant comparisons.

**Table 2:** Matrix of Tukey pairwise probabilities comparing log species richness corrected for area between elevational zones.

	0–249 m	250–499 m	500–749 m	750–999 m	1000–1249 m	1250–1499 m	1500–1749 m
250–499 m	0.000125						
500–749 m	0.000125	0.000125					
750–999 m	0.000125	0.000125	0.995997				
1000–1249 m	0.192009	0.000167	0.000125	0.000125			
1250–1499 m	1.000000	0.000125	0.000125	0.000125	0.095067		
1500–1749 m	0.000125	0.000125	0.000125	0.000125	0.000125	0.000125	
1750–1999 m	0.000125	0.000125	0.000125	0.000125	0.000125	0.000125	0.297001

### Bergmann’s Rule

**Figure 7:**
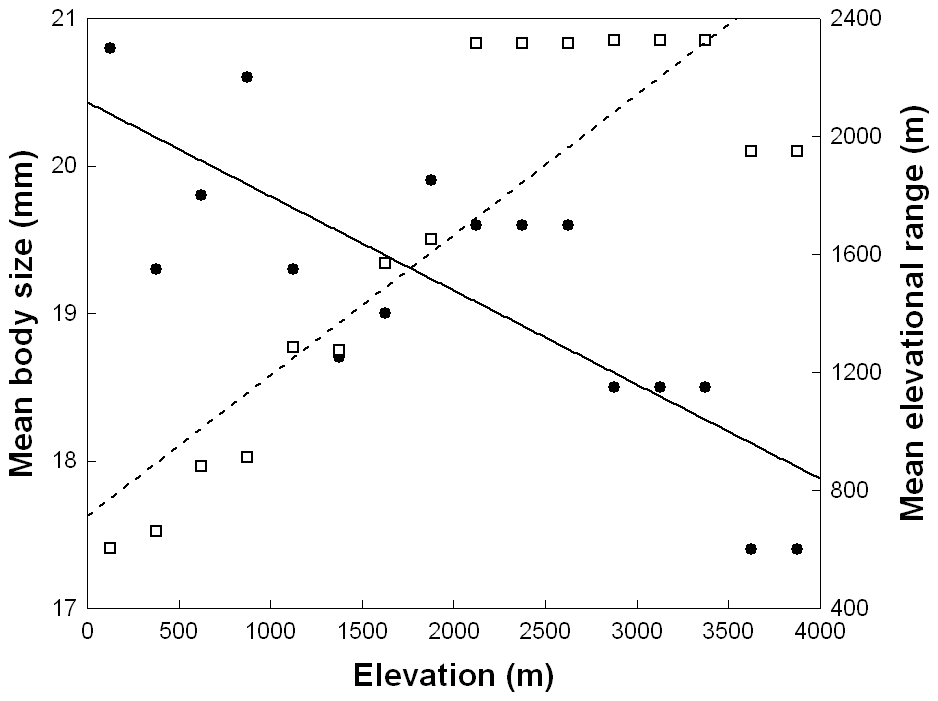
The relationship of mean body size (dots, solid line) and mean elevational range (squares, dashed line) of phanaeine dung beetle assemblages with elevation. Ordinary least squares regression: *R* ^2^ = 0.60, *P*<0.001 for mean body size; *R* ^2^ = 0.76, *P*<0.0001 for mean elevational range.

Mean body size of Bolivian phanaeine species varied from 8.0 mm to 45.5 mm (Table S1). The body size distribution did not differ significantly from a normal distribution (Kolmogorov-Smirnov test: *d* = 0.17, *P*<0.20), although it was slightly right skewed (skew: 1.71±0.38, kurtosis: 3.55±0.74) due to five exceptionally large species (mean body size ≥27 mm), which were recorded exclusively below 1000 m (Table S1). We found no support for Bergmann’s rule. Larger species did not have higher elevational mid-points (Pearson *r* = 0.04, *P* = 0.80 for all phanaeines; *r* = –0.02, *P* = 0.95 for the genus *Coprophanaeus*). In direct contrast to Bergmann’s rule, mean body size per elevational zone showed a pronounced overall decrease with elevation (OLS regression: *R* = –0.78, *P*<0.001 for all phanaeines; *R* = –0.87, *P*<0.01 for the genus *Coprophanaeus*) (Fig. 7). When using only raw species presence data of all phanaeines from the subset of 109 systematically inventoried localities (with localitiy elevation as predictor variable), mean body size also decreased significantly with elevation (OLS regression: *R* = –0.27, *P*<0.01). The relationship remained significant after factoring out the effect of spatial autocorrelation (autoregressive models: geographical distances: *R* = –0.25, *P*<0.01; Gabriel Criterion: *R* = –0.23, *P*<0.05).

### Rapoport’s Rule

The recorded elevational range of species varied from 50 m or less to 2700 m (Table S1), with a mean (± SD) of 664±620 m. Eighteen species (46%) had elevational ranges of 500 m or less, 14 (78%) of which were ecoregional endemics (Table S1). Body size did not have a significant effect on a species’ elevational range (*R* = 0.22, *P* = 0.17 for all phanaeines; *R* = 0.44, *P* = 0.18 for the genus *Coprophanaeus*). We found considerable support for Rapoport’s rule. Species with higher elevational mid-points had greater elevational ranges (Pearson *r* = 0.59, *P*<0.0001 for all phanaeines; *r* = 0.61, *P*<0.05 for the genus *Coprophanaeus*). At the species-assemblage level, mean elevational range increased strongly with elevation (OLS regression: *R* = 0.87, *P*<0.0001 for all phanaeines; *R* = 0.77, *P*<0.05 for the genus *Coprophanaeus*) and remained constantly high above 2000 m, except for a slight drop in the two highest elevational belts (Fig. 7), where only one species was found (Fig. 1). When using only raw species presence data of all phanaeines from the subset of 109 systematically inventoried localities (with localitiy elevation as predictor variable), mean elevational range also increased significantly with elevation (OLS regression: *R* = 0.76, *P*<0.0001). The relationship remained significant after factoring out the effect of spatial autocorrelation (autoregressive models: geographical distances: *R* = 0.76, *P*<0.0001; Gabriel Criterion: *R* = 0.68, *P*<0.001).

## Discussion

To our knowledge this is one of the most comprehensive studies, and one of the first at a regional spatial scale not based on analyses of local elevational transects, on invertebrate biogeographic patterns across an elevational gradient in the Neotropics. In general, our findings confirm the results of most previous studies on insects and other invertebrates. Although phanaeine dung beetle species richness showed a strong overall decrease with elevation, it did not peak in the lowlands, but rather showed a low-elevation hump around 400 m, which became more pronounced and shifted slightly upslope to about 750 m when correcting for area. In general, the elevational richness pattern of ecoregional endemics paralleled that of all species, but the relationship between these variables appears to be curvilinear, providing only partial support for the null hypothesis that species-rich areas are more likely to be centers of endemism by chance alone [52] (see also [53]). The observed increase in the proportion of ecoregional endemics with elevation, particularly so above 1000 m, suggests that in the Andes deterministic factors may also influence patterns of endemism. Our findings directly contradict Bergmann’s rule, showing that high-elevation species assemblages are smaller in mean body size. Rapoport’s rule was strongly supported by our analyses, showing that high-elevation species had broader elevational ranges.

### Species Richness and Endemism

A hump-shaped pattern with maximum richness at some intermediate point of the gradient is the most common elevational richness pattern from local to regional scales and across a wide range of taxonomic groups [2], [9], [16], including mammals [11], [76], birds [3], [8], [12], amphibians [77], insects [31], [78] and plants (e.g., [15], [79], [80], [81]). In the case of Bolivian phanaeines, the low-elevation richness hump at around 400 m (uncorrected) to 750 m (corrected for area) is probably a result of the presence of three species-rich ecoregions, each with a distinctive phanaeine species composition, in this elevational range [51]: the transition zone between southwest Amazonian lowland and humid montane (Yungas) forest in the east Andean foothills; and the Cerrado ecoregion on Precambrian sandstone ridges and plateaus of the Brazilian Shield in eastern Bolivia [59], such as Serranía de Huanchaca in Noel Kempff Mercado National Park [82]. In essence, a foothill overlap of a distinct lowland with a distinct highland phanaeine fauna appears to be responsible for the low-elevation species richness peak as has been shown for birds in central Bolivia [8]. To determine whether this asymmetric pattern is caused by environmental factors, geometric constraints or species’ environmental tolerances, or the interaction of these factors (e.g., [83]), goes beyond the scope of this study.

A general decrease in scarabaeine species richness with elevation was already noted by Lobo and Halffter [84]. Overall, the pronounced elevational decrease (especially between 500 m and 2000 m) of phanaeine species richness in Bolivia is in accordance with studies on a range of plant, invertebrate and vertebrate taxa that demonstrated the combined influence of temperature (energy) and water availability on species diversity (e.g., [85], [86]), particularly along elevational gradients [11], [12]. However, due to their Gondwanaland origin [87], [88], scarabaeine dung beetles are a group mostly adapted to warm or warm-temperate conditions [89], and rather than contemporary climate, this may have constrained the current distribution of most species to lower elevations (see [47]) in accordance with the niche conservatism hypothesis (e.g., [90], [91]). In addition to these ultimate causes of scarabaeine and phanaeine elevational richness patterns, proximate causes are likely to be related to biomass and richness patterns of the vertebrate species they have coevolved with [47], [51], [87].

No regional-scale studies (using geographically defined regions rather than local transects as units of analysis) on elevational gradients of Neotropical dung beetle diversity exist for comparison with our data set. Several analyses of local transects that did not control for the effect of area (primarily from Colombia; [10], [92], [93]) showed mid-elevation peaks of scarabaeine species richness between 300 m and 1300 m. The pattern is most consistent and pronounced on the eastern (Orinoquían and Amazonian) slope of the eastern Cordillera of the Colombian Andes, where species richness peaked at about 1300 m along each of five elevational transects [92], although areas below 1000 m were not sampled. Escobar et al. [92] attributed this hump-shaped species richness pattern to the contact and mixing of faunas with different climatic tolerances and, probably, different lineages and history from the Amazonian and Orinoquían lowlands.

However, not all studies documented hump-shaped relationships between species richness and elevation for Neotropical dung beetles. On the Pacific slope of the western Colombian Andes [10] and on the east Andean slope in southeast Peru [94] species richness decreased virtually linearly with elevation, although the effect of area was not controlled for in these studies. When pooling data from 12 local-scale elevational transects along the eastern slopes of the tropical Andes, Larsen et al. [78] found yet another pattern: an exponential decrease of dung beetle species richness with elevation. This array of observed relationships indicates the need for further studies to develop a general understanding of elevational species richness patterns of Neotropical dung beetles and their underlying causes as well as any latitudinal or regional variation that may exist in these patterns.

A parallel decrease of total and endemic species richness with elevation also was found for scarabaeine dung beetles along local transects in the Colombian Andes [92], despite a different definition of ‘endemic’ species. Escobar et al. [92] reported a significant correlation between the number of geographically restricted species and total species richness per site, although they did not examine whether the relationship may be curvilinear. Elevational patterns in the proportion of endemics also were not examined by Escobar et al. [92]. This decrease in the number of endemic South American dung beetle species with elevation contrasts in part with patterns found in a range of taxonomic groups such as birds ([95] and references therein) and most small mammals ([96] and references therein), where endemism generally increases with elevation in both absolute and relative terms, peaking at or near the Andean timberline ecotone. However, a more refined analysis of elevational endemism patterns in dung beetles based on estimates of actual range size (once these are available) rather than endemism proxies may reveal somewhat different patterns.

### Bergmann’s Rule

Bergmann’s rule was not supported at the species level. At the species-assemblage level, decreasing mean body size of phanaeine dung beetles with elevation contradicted Bergmann’s rule. Previous studies on body size changes along elevational gradients of Neotropical butterflies [25], geometrid moths [17] and tephritid flies [28] did not find support for Bergmann’s rule either. Brehm and Fiedler [17] argued that geographic body size patterns in Neotropical lepidopterans are mainly characterized by taxonomic idiosyncrasies, and that geometrid moths in particular do not require large bodies to maintain a certain flight temperature because of their relatively low thoracic temperatures during flight. By contrast, Kubota et al. [28] hypothesized that heat-gaining capacity may be more important for insects at high elevations than heat conservation, and that small insects may warm up more quickly than bigger ones. Due to their more rapid and pronounced warming and chilling, smaller insect species should be more thermally tolerant, and this in turn would enable them to occur at higher elevations [28].

Whether any of these explanations apply to phanaeine dung beetles, or if elevational body size patterns of phanaeines are primarily influenced by the quantity and quality of available food resources, remains to be tested. It certainly seems plausible that the absence of very large vertebrates such as the South American tapir (*Tapirus terrestris*) from Andean forests may contribute to smaller mean body size at higher elevations. Nonetheless, physiological studies on thermoregulation in 24 dung beetle species from Mexico, Kenya and Spain [97], [98] may provide another working hypothesis for why Bolivian phanaeines do not conform to Bergmann’s rule. Both studies documented a high correlation of body temperature with body mass. Larger species show strong endothermy, elevating and maintaining their body temperature at levels well above ambient temperature during flight and dung ball making and rolling, whereas smaller species (<1.9 g) are unable to do so, resulting in body temperatures that are very similar to ambient temperatures [98]. Therefore, because endothermic activity of larger dung beetle species can be expected to become increasingly costly energetically with increasing elevation (and a concommittant decrease in ambient temperatures), such species may be limited to low elevation areas.

### Rapoport’s Rule

As predicted by Rapoport’s elevational rule [26], phanaeine species and species assemblages at higher elevations had broader elevational ranges. The proposed underlying mechanism of this rule is that species at higher elevations can tolerate greater climatic variability and therefore have larger elevational ranges, because high-elevation climates are more variable and show a greater magnitude of extremes than low-elevation climates. Thus, high-elevation phanaeine species can be expected to have broader thermal tolerances than low-elevation species. Gaston and Chown [27] provided physiological evidence for this prediction from southern Africa, where the thermal tolerance range (and the elevational range) of dung beetles increased with elevation. There also is general support for Rapoport’s rule among other arthropod taxa, including butterflies in the southwestern United States [32] and Spain [34], geometrid moths in Costa Rica [31], ants in the western United States [33], tephritid flies [28] and opilionid arachnids [30] in southeastern Brazil and gnaphosid spiders in Greece [35].

### Conservation Implications

Tropical insects may be particularly sensitive and vulnerable to global climate change [55], [56], [57] and deforestation [99]. In response to a warming climate, tropical species are shifting their geographic ranges toward cooler temperatures at higher elevations [100], [101], [102]. In contrast to temperate regions, elevational temperature gradients in the tropics are vastly steeper (>1000 times) than latitudinal temperature gradients, making upslope range shifts the more likely response of tropical species to climate warming [55]. Habitat fragmentation and deforestation can likewise lead to warmer, drier microclimates and concomitant up-slope range shifts of species [99]. This upslope displacement of species distributions bears several biogeographic consequences that pose serious conservation problems, including mountaintop extinctions [101], range-shift gaps (spatial discontinuity between current and projected future range), and lowland biotic attrition, *i.e.*, the net loss of species richness in the tropical lowlands from upslope range shifts and lowland extinctions [55]. Indeed, experimental studies show that tropical lowland insects are currently living very close to their optimal temperature, so that continued climate warming would lead to a decrease in their fitness and, eventually, extinction [56].

Analyses of biogeographic patterns across elevational gradients such as Rapoport’s rule therefore have the potential to provide important information for the identification of conservation priorities in the tropics. Among Bolivian phanaeines, most Andean species are unlikely to be threatened by mountaintop extinctions or range-shift gaps due to their broad elevational ranges and accordingly high thermal tolerance combined with low species richness above 2000 m. Rather, low-elevation species with narrow range amplitudes and presumably low thermal tolerance may be expected to be at much greater risk of extinction, potentially leading to lowland biotic attrition as predicted by Colwell et al. [55]. In accordance with Hamel-Leigue et al. [51], who examined ecoregional diversity patterns of Bolivian phanaeines, mountaintop extinctions may constitute an imminent threat to cerrado endemics on low, isolated mountain ranges in eastern Bolivia such as the Serranía de Huanchaca (ca. 500–800 m). This region is already experiencing significant negative impacts of anthropogenic change, which have lead to small mammal population crashes in savanna grasslands [103]. Range-shift gaps may also be of serious concern as 18 phanaeine species (46%) have known elevational ranges of 500 m or less, 14 of which are ecoregional endemics (Table S1). With two exceptions (*Coprophanaeus caroliae* Edmonds, 2008; *Phanaeus lecourti* Arnaud, 2000), these species occur only below 1000 m. Because much of the ecoregional endemism is concentrated below 1000 m, low-elevation regions are an even greater conservation priority under climate change.

In conclusion, as predicted by the climatic variability hypothesis [27], endemic species of low-elevation ecoregions may be the phanaeines most vulnerable to climate change. The situation is perhaps most critical for cerrado endemics on isolated mountain ranges in Santa Cruz Department. Theoretically, those species can escape mountaintop extinctions only by long-distance dispersal of about 400–500 km to the east Andean foothills, which may not provide suitable habitat for them. Even if such suitable habitat existed, whether it harbours appropriate vertebrate dung resources for cerrado phanaeines may be equally important for the successful establishment of founder populations. Given that large mammals are less sensitive to climate warming than insects, dung beetles may shift their ranges more rapidly than the hosts they depend upon. A thorough understanding of the degree of vertebrate host specificity of cerrado species, as well as the distribution and conservation status of medium and large mammals in Bolivia (see Wallace et al. [104] for recent advances), will thus be crucial. Confirming predictions based on the climatic variability hypothesis [27] through experimental studies on the thermal tolerance of low-elevation ecoregional endemics will be equally important for devising successful phanaeine conservation strategies in a changing world.

## Supporting Information

Table S1 **Elevational range and mean body size of phanaeine dung beetles in Bolivia.**
(DOC)

Table S2 **Number of phanaeine dung beetle species (*N* = 89) and surface area of South American countries.**
(DOC)

Table S3 **Number of 0.08333 arc degree cells and area of 16 elevational zones in Bolivia.**
(DOC)

Table S4 ***z*-values from Rahbek’s [3]fig. 2 for South American land birds scaled down to the narrower bandwidth of the present study using spline-interpolated fitted curves.**
(DOC)
