# Supplementary material for: Elevational Distribution and Conservation Biogeography of Phanaeine Dung Beetles (Coleoptera: Scarabaeinae) in Bolivia
Source: PLoS One. 2013 May 22;8(5):e64963. doi: 10.1371/journal.pone.0064963 (PMC3661563; doi:10.1371/journal.pone.0064963)
Supplement: Table S1 — Elevational range and mean body size of phanaeine dung beetles in Bolivia. (DOC) [file pone.0064963.s001.doc]

# Table S1. Elevational range and mean body size of phanaeine dung beetles in Bolivia.

| **Species** | **Elevational**  **range (m)** | **Mean body size (mm)** | **Specimens measured^1^** |
| --- | --- | --- | --- |
| *Bolbites onitoides** | 300-800 | 16.7 | 6 |
| *Coprophanaeus acrisius* | 150-900 | 27.0 | 2 |
| *C. bonariensis* | 200-950 | 31.9 | 7 |
| *C. caroliae** | 1250-1350 | 19.5 | 1 |
| *C. cyanescens* | 200-900 | 22.0 | 12 |
| *C. ensifer* | 200-800 | 45.5 | 1 |
| *C. ignecinctus** | 700-1800 | 19.6 | 38 |
| *C. lancifer** | 100-300 | 39.6 | 61 |
| *C.* *magnoi* * | 150-200 | 20.4 | 7 |
| *C. pessoai** | 250 | 17.5 | 0 |
| *C. suredai** | 100-150 | 18.5 | 0 |
| *C. telamon* | 100-1150 | 21.5 | 61 |
| *Dendropaemon* nr. *bahianus** | 300 | 10.3 | 0 |
| *D. denticollis** | 150-400 | 8.0 | 1 |
| *D. pauliani** | ~300-400 | 13.0 | 0 |
| *D. viridis** | 700-800 | 18.0 | 0 |
| *Diabroctis mimas* | 100-1300 | 24.1 | 17 |
| *D. mirabilis** | 700-800 | 18.5 | 2 |
| *Gromphas aeruginosa* | 200-500 | 13.7 | 6 |
| *G. lacordairei* | 150-600 | 12.9 | 39 |
| *Oruscatus davus* | 2050-4000 | 17.4 | 24 |
| *Oxysternon conspicillatum* | 100-1150 | 23.7 | 60 |
| *O. lautum* | 150-200 | 22.5 | 60 |
| *O. palaemon* | 200-800 | 14.3 | 24 |
| *O. silenus* | 100-1150 | 16.8 | 64 |
| *O. spiniferum* | 250-1300 | 11.0 | 1 |
| *O. striatopunctatum** | 450-800 | 12.0 | 2 |
| *Phanaeus alvarengai* | 100-450 | 17.5 | 3 |
| *P. bispinus* | 100-800 | 14.8 | 11 |
| *P. cambeforti* | 100-700 | 14.2 | 6 |
| *P. chalcomelas* | 100-1000 | 14.7 | 49 |
| *P. kirbyi* | 200-900 | 15.2 | 13 |
| *P. lecourti** | 1300-1800 | 18.6 | 8 |
| *P. meleagris* | 350-1600 | 15.5 | 90 |
| *P. melibaeus** | 450 | 14.5 | 2 |
| *P. palaeno* | 200-900 | 15.5 | 1 |
| *Sulcophanaeus batesi* | 600-3300 | 19.6 | 107 |
| *S. faunus* | 100-900 | 33.4 | 31 |
| *S. imperator* | 300-2600 | 21.9 | 7 |

Length measured from pygidium to anterior margin of clypeus. Ecoregional endemics are marked by “*” (see text for details).

^1^ Number of specimens measured by the authors. For *N* > 5, mean body size values are based exclusively on those measurements. For the remaining species data were supplemented with values from the literature (see text for details).
