# Supplementary material for: Elevational Distribution and Conservation Biogeography of Phanaeine Dung Beetles (Coleoptera: Scarabaeinae) in Bolivia
Source: PLoS One. 2013 May 22;8(5):e64963. doi: 10.1371/journal.pone.0064963 (PMC3661563; doi:10.1371/journal.pone.0064963)
Supplement: Table S2 — Number of phanaeine dung beetle species (N = 89) and surface area of South American countries. (DOC) [file pone.0064963.s002.doc]

# Table S2. Number of phanaeine dung beetle species (*N* = 89) and surface area of South American countries.

| **Country** | **Number of species** | **Surface area (km^2^)** |
| --- | --- | --- |
| Argentina | 16 | 2 780 400 |
| Bolivia | 35 | 1 098 581 |
| Brazil | 52 | 8 514 877 |
| Colombia | 29 | 1 141 748 |
| Ecuador | 19 | 283 561 |
| French Guiana | 15 | 83 534 |
| Guyana | 8 | 214 970 |
| Paraguay | 18 | 406 752 |
| Peru | 26 | 1 285 216 |
| Suriname | 8 | 163 821 |
| Uruguay | 5 | 176 215 |
| Venezuela | 26 | 916 445 |

No species have been reported for Chile.

Phanaeine genera included: *Bolbites*, *Coprophanaeus*, *Diabroctis*, *Gromphas*, *Homalotarsus*, *Megatharsis*, *Oruscatus*, *Oxysternon*, *Phanaeus*, *Sulcophanaeus*, *Tetramereia*. The genus *Dendropaemon* was excluded due to species-level taxonomic uncertainties.
