# Supplementary material for: Elevational Distribution and Conservation Biogeography of Phanaeine Dung Beetles (Coleoptera: Scarabaeinae) in Bolivia
Source: PLoS One. 2013 May 22;8(5):e64963. doi: 10.1371/journal.pone.0064963 (PMC3661563; doi:10.1371/journal.pone.0064963)
Supplement: Table S3 — Number of 0.08333 arc degree cells and area of 16 elevational zones in Bolivia. (DOC) [file pone.0064963.s003.doc]

# Table S3. Number of 0.08333 arc degree cells and area of 16 elevational zones in Bolivia.

| **Elevational zone (m)** | **Number of cells** | **Area (km^2^)** |
| --- | --- | --- |
| 79-249 | 5123 | 422 873 |
| 250-499 | 2834 | 236 764 |
| 500-749 | 466 | 39 397 |
| 750-999 | 284 | 24 294 |
| 1000-1249 | 264 | 22 848 |
| 1250-1499 | 214 | 18 734 |
| 1500-1749 | 216 | 19 125 |
| 1750-1999 | 164 | 14 685 |
| 2000-2249 | 167 | 15 121 |
| 2250-2499 | 162 | 14 830 |
| 2500-2749 | 181 | 16 750 |
| 2750-2999 | 171 | 15 996 |
| 3000-3249 | 182 | 17207 |
| 3250-3499 | 195 | 18 631 |
| 3500-3749 | 193 | 18 633 |
| 3750-3999 | 184 | 17 948 |

The high-elevation Altiplano ecoregion Southern Puna and the subecoregion Humid Puna of south-west Bolivia were excluded as they do not appear to host any phanaeine species (based on limited collecting work in these regions by the authors; see [47]).
