# Supplementary material for: Elevational Distribution and Conservation Biogeography of Phanaeine Dung Beetles (Coleoptera: Scarabaeinae) in Bolivia
Source: PLoS One. 2013 May 22;8(5):e64963. doi: 10.1371/journal.pone.0064963 (PMC3661563; doi:10.1371/journal.pone.0064963)
Supplement: Table S4 — z-values from Rahbek’s [3]fig. 2 for South American land birds scaled down to the narrower bandwidth of the present study using spline-interpolated fitted curves. (DOC) [file pone.0064963.s004.doc]

# Table S4. *z*-values from Rahbek’s [3] fig. 2 for South American land birds scaled down to the narrower bandwidth of the present study using spline-interpolated fitted curves.

| **Elevational zone (m)** | **Panel A** | **Panel B** | **Panel C** |
| --- | --- | --- | --- |
| 250-499 | 0.175 | 0.182 | 0.111 |
| 500-749 | 0.131 | 0.134 | 0.144 |
| 750-999 | 0.122 | 0.119 | 0.143 |
| 1000-1249 | 0.156 | 0.151 | 0.103 |
| 1250-1499 | 0.173 | 0.183 | 0.091 |
| 1500-1749 | 0.163 | 0.196 | 0.116 |
| 1750-1999 | 0.165 | 0.206 | 0.138 |
| 2000-2249 | 0.193 | 0.22 | 0.139 |
| 2250-2499 | 0.227 | 0.235 | 0.132 |
| 2500-2749 | 0.250 | 0.245 | 0.13 |
| 2750-2999 | 0.259 | 0.251 | 0.135 |
| 3000-3249 | 0.261 | 0.255 | 0.144 |
| 3250-3499 | 0.260 | 0.258 | 0.155 |

Elevational zones in Rahbek [3]: 0-500 m, 500-1000 m, 1000-1500 m, 1500-2000 m, 2000-3000 m, 3000-4000 m. See text for details on downscaling procedure. Panel A = all countries except Chile (country grouping A); panel B = all countries except Chile and Brazil (country grouping B); panel C = only the tropical Andes countries Bolivia, Colombia, Ecuador, Peru (country grouping C).
